# An absorptive coding metasurface for ultra-wideband radar cross-section reduction

**DOI:** 10.1038/s41598-024-63260-z

**Published:** 2024-05-29

**Authors:** Baoqin Lin, Wenzhun Huang, Jianxin Guo, Zuliang Wang, Kaibo Si, Hongjun Ye

**Affiliations:** School of Electronic Information, Xijing University, Xi’an, China

**Keywords:** Applied physics, Electronics, photonics and device physics, Plasma physics

## Abstract

In this paper, an absorptive coding metasurface (ACM) is proposed for ultra-wideband radar cross section (RCS) reduction, the design process is presented in detail, in which a lossy polarization conversion metasurface (PCM) is proposed at first. The lossy PCM is an anisotropic resistive structure with both polarization conversion and absorption performances, so that its co-polarization reflection coefficients under *u*- and *v*-polarized incidences can be kept at less than − 10 dB in magnitude in the frequency range from 7.5 to 45.2 GHz. Though the magnitude of the cross-polarization reflection coefficient cannot be very small only due to the absorption, its phase will be changed by nearly 180° when the unit-cell structure of the lossy PCM is rotated by 90°. Thus, the lossy PCM can be used as one of the two types of lossy coding elements for an ACM when its unit-cell structure is rotated by 90° or not. Based on the lossy PCM, an ACM is proposed. The simulation and experimental results show that the ACM has an excellent RCS reduction performance under arbitrary polarized incidence, it can achieve effective RCS reduction under normal incidence in the ultra-wide frequency band from 7.4 to 45.5 GHz with a ratio bandwidth (*f*_H_/*f*_L_) of 6.15:1; moreover, an ultra-wideband RCS reduction can still be achieved when the incident angle is increased to 45°, which indicates that the ACM has good stealth performance under the detection of various radars working in X, Ku, K and Ka bands, it is very practical.

## Introduction

Radar cross section (RCS) is a key factor in evaluating the radar visibility of a stealth target, so the RCS of various stealth targets should be reduced as much as possible, how to fully reduce the RCS of various stealth targets is always a hot research topic in the field of electromagnetics. Metasurface is an artificial composite surface composed of an array of sub-wavelength resonant unit-cell structures. Through the reasonable design of the unit-cell structure and the arrangement, almost all the basic properties of electromagnetic (EM) wave, such as magnitude, phase and polarization, can be tailored by using various metasurfaces. Due to the super performance, numerous different types of metasurfaces, such as absorbing-type metasurface^1–6^ and coding metasurface^7^, have been proposed to reduce the RCS of various radar stealth targets in the recent decade.

The unit cell structure of an absorbing-type metasurface usually contains a high-lossy dielectric layer, or several resistive patches or lumped resistors. Thus, under EM wave incidence, such an absorbing-type metasurface can convert the EM energy into heat energy around the resonant frequency of the unit-cell structure, and achieve RCS reduction by means of absorption. However, the absorbing-type metasurface usually can’t achieve ultra-wideband absorption because the Q value of the unit-cell resonance is difficult to be very low when the total thicknesses is thin relative to the working wavelength. In addition, a coding metasurface is composed of numerous coding elements that can be divided into N types, and the reflection phases in different types of coding elements will differ greatly under same incidence, which will cause their reflected waves to cancel each other. Thus, through the appropriate arranging of these types of coding elements, the composed coding metasurface can diffuse its reflected waves in various directions due to phase cancellation, and achieve effective RCS reduction by means of diffusion. In recent years, diffusion has become the most common means of achieving RCS reduction, a large number of different coding metasurfaces have been proposed^8–24^, and the coding metasurfaces proposed in Ref.^19–24^ could all achieve ultra-wideband RCS reduction with a fractional bandwidth wider than 100%. Though the absorption and diffusion are the two different means of achieving RCS reduction, the two means can be carried out simultaneously based on a proper lossy metasurface. Thus, in recent years, a number of absorptive coding metasurfaces (ACMs) have been proposed^25–38^, which could achieve RCS reduction by means of both absorption and diffusion.

In this paper, in order to achieve RCS reduction with a much wider band, a novel ACM is proposed. The design process is presented in detail, in which an ultra-wideband lossy polarization conversion metasurface (PCM) is proposed at first. The lossy PCM can achieve linear polarization conversion and absorption to make the magnitude of its co-polarization reflection coefficients under UP and VP incidences very small in an ultra-wide frequency band; in addition, the phase of its cross-polarization reflection coefficients can be changed by nearly 180° when its unit-cell structure is rotated by 90°. So, the PCM can be used as one of the two types of lossy coding elements of a 1-bit ACM when its unit-cell structure is rotated by 90° or not, and an ultra-wideband ACM is proposed based on the lossy PCM. The simulation and experimental results show that the proposed ACM can achieve ultra-wideband RCS reduction under arbitrary polarization incidence with an incident angle less than 45°. Compared with many previous designs, the ACM exhibits much wider bandwidth, so it is very practical.

## Design and simulation

**Figure 1:**
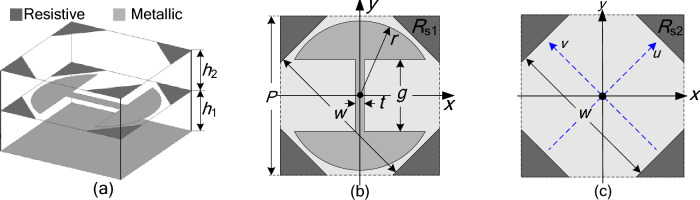
Unit cell structure of the proposed lossy PCM: (**a**) the 3D view; (**b**) the middle patterned layer; (**c**) the upper patterned layer.

To design a coding metasurface, several types of coding elements with different reflection phases should be constructed firstly. In this work, in order to construct two types of lossy coding elements for a 1-bit ACM, a lossy PCM is proposed, which consists of a metallic patch array and two resistive patch arrays mounted on a grounded dual-layer dielectric substrate. One unit cell structure of the lossy PCM is shown in Fig. 1, it is indicated that the metallic patch in the unit cell is an anisotropic structure which is symmetrical about both *x*- and *y*-axes, so a phase difference $\Delta \varphi_{{x{\text{y}}}}$ will exist between the reflection coefficients *r*_*xx*_ and* r*_*yy*_ when the lossy PCM is illuminated by *x*-polarized (XP) and *y*-polarized (YP) waves. If the phase difference $\Delta \varphi_{{x{\text{y}}}}$ is close to 180°, according to the theoretical derivation shown in Ref.^39^, we can know that the lossy PCM will achieve linear polarization conversion under *u*-polarized (UP) and *v*-polarized (VP) incidences; moreover, the phase of the cross-polarization reflection coefficients *r*_*vu*_ and* r*_*uv*_ will be changed by nearly180° when the unit-cell structure of the lossy PCM is rotated by 90°. In addition, the two resistive patches in the unit cell will introduce a certain ohmic loss, so the lossy PCM will have a certain absorbing performance. In this way, it is known that if the structural parameters of the lossy PCM are all appropriate, the lossy PCM can achieve both linear polarization conversion and absorption, its co-polarization reflection coefficients *r*_*uu*_ and* r*_*vv*_ under UP and VP incidences can be very small in magnitude in an ultra-wide frequency range; moreover, the phase of cross-polarization reflection coefficients *r*_*vu*_ and* r*_*uv*_ will be changed by nearly 180° when its unit-cell structure is rotated by 90°. Thus, the lossy PCM can be used as one of the two types of lossy coding elements for a 1-bit ACM when its unit-cell structure is rotated by 90° or not. In order to achieve the purpose, through repeated simulations, the structural parameters of the unit-cell structure are finally chosen as follows: *h*_1_ = 1.95 mm, *h*_2_ = 1.95 mm, *P* = 6.00 mm, *r* = 2.80 mm,* g* = 2.80 mm, *t* = 0.55 mm, *w* = 6.00 mm, *R*_s1_ = 95 Ω/m^2^, *R*_s2_ = 190 Ω/m^2^; in addition, the two dielectric layers are both chosen as a PTFE with a relative permittivity of *ε*_*r*_ = 1.8 and a loss tangent of tan δ = 0.0012.

**Figure 2:**
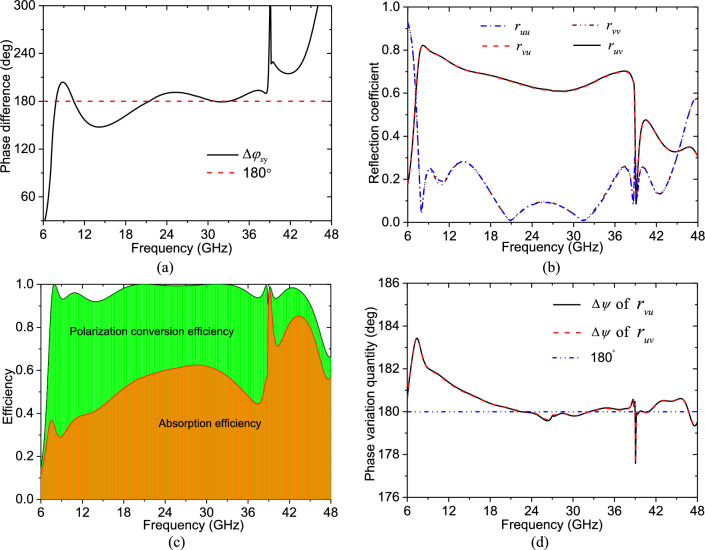
Simulation results of the proposed lossy PCM: (**a**) the phase difference $\Delta \varphi_{{x{\text{y}}}}$; (**b**) the magnitude of *r*_*uu*_, *r*_*vu*_,* r*_*vv*_ and* r*_*uv*_; (**c**) the efficiencies of polarization conversion and absorption; (**d**) the phase variation quantities $\Delta \psi$ of *r*_*vu*_ and *r*_*uv*_ when the unit cell structure is rotated by 90°.

In order to analyse the performance of the lossy PCM, we have simulated it under XP and YP incidences firstly by using Ansoft HFSS. The obtained simulation results are shown in Fig. 2a, it is indicated that the phase difference $\Delta \varphi_{{x{\text{y}}}}$ of the lossy PCM is close to 180° in the ultra-wide frequency range from 7.5 to 44.0 GHz except for near the frequency point 39.1 GHz, which implies that the lossy PCM can achieve linear polarization conversion in this frequency range under both UP and VP incidences. To verify the prediction, the lossy PCM has been simulated under UP and VP incidences, the simulation results, shown in Fig. 2b, indicate that the magnitude of *r*_*vu*_ and* r*_*uv*_ is much larger than that of *r*_*uu*_ and* r*_*vv*_ in the frequency range from 7.5 to 44.0 GHz except for near the frequency point 39.1 GHz, which shows that the anticipated linear polarization conversion is achieved. Moreover, due to the absorption, the max magnitude of *r*_*vu*_ and* r*_*uv*_ is only 0.81, in particular, at the frequency point 39.1 GHz where no linear polarization conversion is achieved, the magnitude of the co-polarization reflection coefficients *r*_*vv*_ and *r*_*uu*_ is still only 0.19. These simulation results indicate that the lossy PCM can achieve both polarization conversion and absorption, the polarization conversion efficiency calculated by formula $\left| {r_{vu} } \right|^{2}$ or $\left| {r_{uv} } \right|^{2}$, together with the absorption efficiency calculated by formula $1- \left| {r_{vu} } \right|^{2} - \left| {r_{uu} } \right|^{2}$ or $1 - \left| {r_{uv} } \right|^{2} - \left| {r_{vv} } \right|^{2}$, is shown in Fig. 2c, it is indicated that the co-polarized reflection efficiency of the lossy PCM is very small in an ultra-wide frequency band due to the polarization conversion and absorption. In addition, Fig. 2b shows that the magnitude of *r*_*uu*_ and* r*_*vv*_ can be kept at less than 0.316 (− 10 dB) in the frequency range from 7.5 to 45.2 GHz. Furthermore, to verify that the phases of *r*_*vu*_ and* r*_*uv*_ will be changed by rotating the unit-cell structure, the lossy PCM is also simulated when its unit-cell structure is rotated by 90°. Thus, the phase variation quantities $\Delta \psi$ of *r*_*vu*_ and *r*_*uv*_ due to the rotation of the unit-cell structure are obtained, which are shown in Fig. 2d, it is indicated that the phase variation quantities $\Delta \psi$ of *r*_*vu*_ and *r*_*uv*_ are both basically equal to 180° at all frequencies.

**Figure 3:**
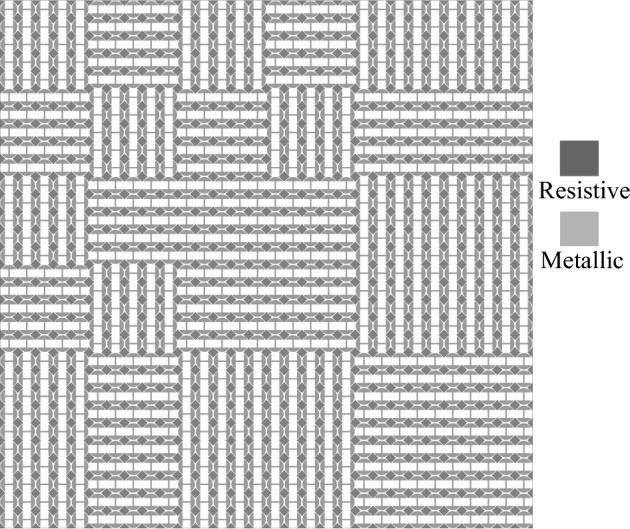
Schematic diagrams of the proposed ACM.

Now, according to the simulation results shown in Fig. 2, we can know that the lossy PCM can be used as one of the two ypes of lossy coding elements for a 1-bit ACM when its unit-cell structure is rotated by 90° or not. Thus, based on the lossy PCM, we propose a 1-bit ACM, which consists of 6 × 6 coding elements, and each coding element is composed of 5 × 5 identical sub-unit cells. In addition, in order to achieve ultra-wideband RCS reduction, these coding elements are arrayed according to a proper coding sequence obtained through repeated simulations. The final proposed ACM is shown in Fig. 3.

**Figure 4:**
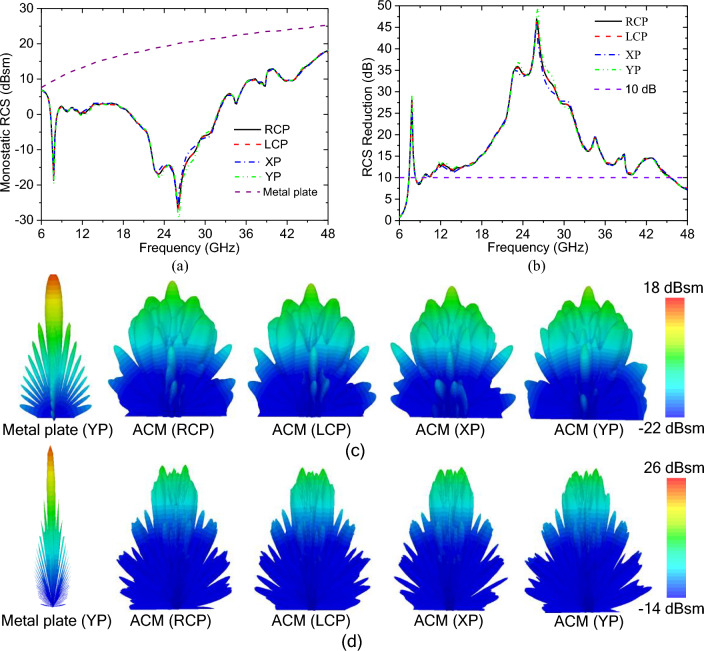
Simulation results of the ACM and the metallic plate under normal incidences with different polarizations: (**a**) the monostatic RCS; (**b**) the monostatic RCS reduction of the ACM; and the 3D far-field scattering patterns at 15.0 GHz (**c**) and 35.0 GHz (**d**).

According to the design process of the ACM, we can know that the ACM would achieve ultra-wideband RCS reduction under both UP and VP incidences. Because an arbitrary polarized wave can be regarded as a composite wave composed of a pair of UP and VP ones, the ultra-wideband RCS reduction would be achieved under arbitrary polarized incidences. In order to verify the theoretical prediction, the ACM, together with a pure metallic plate with the same size, has been simulated under right-handed circular-polarized (RCP), left-handed circular-polarized (LCP), XP and YP incidences. The obtained simulation results, the monostatic RCSs of the ACM and the metallic plate, are shown in Fig. 4a, in which it is indicated that the monostatic RCS of the ACM is almost the same under different polarized incidences, however, compared with that of the metallic plate, the monostatic RCS of the ACM is significantly reduced. In addition, in Fig. 4b, it is shown that the monostatic RCS reductions of the ACM under these incidences can all be kept at more than 10.0 dB in the ultra-wide frequency band from 7.4 to 45.5 GHz except for 8.3 to 9.4 GHz, and the minimum monostatic RCS reduction in the frequency range of 8.3–9.4 GHz is still more than 8.4 dB, which indicates that the ACM can realize ultra-wideband monostatic RCS reduction under arbitrary polarized incidences, the bandwidth ratio is equal to 6.15:1, and the fractional bandwidth reaches up to 144.1%. Furthermore, to show the far-field scattering characteristics of the ACM under these incidences, the three-dimensional (3D) far-field scattering patterns at the frequencies of 15.0 and 35.0 GHz are shown in Fig. 4c and d, respectively. It is indicated that compared with the strong specular reflection of the pure metal plate, the ACM can diffuse its scattering wave into various directions, which shows that the ACM can achieve ultra-wideband diffusion-like scattering, and greatly reduce its monostatic and maximum bistatic RCS simultaneously. In conclusion, according to the simulation results shown in Fig. 4, it is well known that the ACM has excellent RCS reduction performance, moreover, its polarization sensitivity is very low, it can achieve ultra-wideband RCS reduction in the frequency band of 7.4–45.5 GHz under normal incidence with arbitrary polarization.

**Figure 5:**
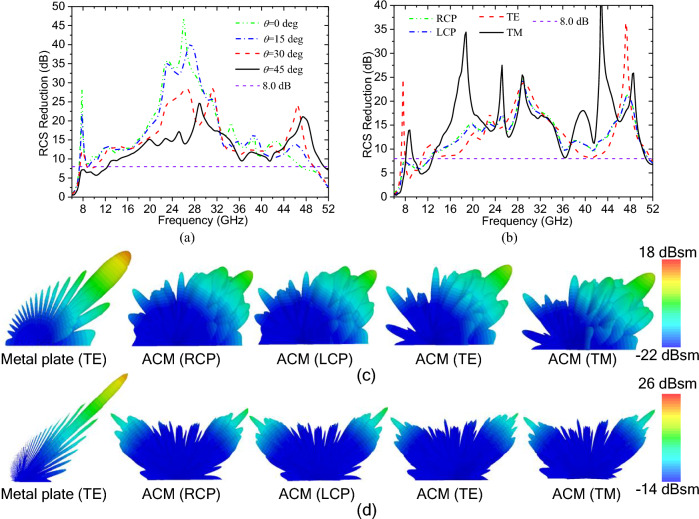
Simulation results of the ACM under oblique incidences: (**a**) the specular RCS reduction under RCP incidence with different incident angles; (**b**) the specular RCS reduction under different polarized incidences with an incident angle of 45°; and the 3D RCS scattering patterns at 15.0 GHz (**c**) and 35.0 GHz (**d**).

Furthermore, to analyse the angular stability, the ACM, together with the pure metallic plate, has been simulated under RCP, LCP, TE and TM oblique incidences. The obtained simulation results, the specular RCS reduction of the ACM relative to the pure metal plate, are shown in Fig. 5a and b. In Fig. 5a, it is shown that when the incident angle θ is increased to 45°, the specular RCS reduction of the ACM under RCP incidence can still be kept at more than 8.0 dB in the ultra-wide frequency band of 12.4–51.2 GHz. In addition, Fig. 5b indicates that when the incident angle is set as 45°, although the RCS reductions under different polarized incidences are not the same, they can all be kept at more than 8.0 dB in the frequency range from 13.4 to 50.4 GHz. Furthermore, the 3D far-field scattering patterns at the frequencies of 15.0 and 35.0 GHz under different polarized incidences with an incident angle of 45° are shown in Fig. 5c and d respectively, it is indicated that the ACM can still achieve ultra-wideband diffusion-like scattering under all these oblique indicences. These simulation results show that the ACM has good angular stability under arbitrary polarized incidences.

## Experimental verification

**Figure 6:**
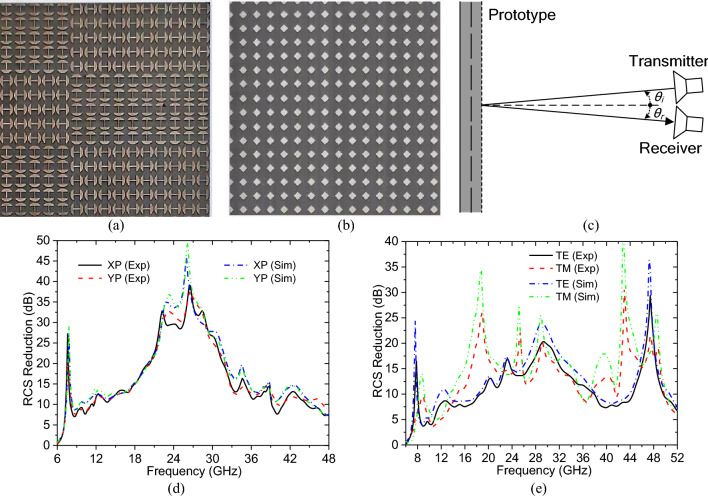
Experimental prototype and experimental results: (**a**) one part of the metallic patch array on the grounded dielectric substrate; (**b**) one part of the resistive patch arrays on the upper dielectric layer; (**c**) the schematic of the measurement setup; (**d**) the monostatic RCS reduction under XP, YP normal incidences; (**e**) the specular RCS reduction under TE, TM incidences with an incident angle of 45°.

Finally, to experimentally verify the performance of the ACM, one experimental prototype has been fabricated and measured. In the fabricating process, the metallic patch array was printed on the grounded dielectric substrate by printed circuit board (PCB) technique, it is shown in Fig. 6a, and the two resistive patch arrays were screen-printed on the front and back sides of the upper dielectric layer using carbon resistive paste, the upper one is shown in Fig. 6b, then the whole structure was fabricated by hot pressing the ground dielectric substrate and the upper dielectric layer together. We have measured the far-field reflection of the experimental prototype and a pure metal plane with the same size under identical experimental conditions. These measurements were all carried out in a microwave anechoic chamber, and the schematic illustration of the measurement setup is shown in Fig. 6c, in which the two identical standard-gain horn antennas as transmitter and receiver have been connected to the two ports of an Agilent E8363B network analyzer. When the incident angle *θ*_*i*_ and the reflection angle *θ*_*r*_ were both set to 3° or 45°, the experimental prototype and the metallic plate have been measured under normal or oblique incidence. However, the incident wave was always a LP one because no ultra-wideband CP antenna exists in common experimental equipments. According to the ratio between the directly measured results of the experimental prototype and the metal plane, the monostatic and specular RCS reduction values of the ACM relative to the metal plate were obtained, which are shown in Fig. 6d and e. It is indicated that the experimental results under the normal and oblique incidences are both in reasonable agreement with the above simulation results except for a slight deviation caused by fabrication errors and measurement tolerance. Thus, the RCS reduction performance of the ACM is verified furtherly. Now, it is determined that the ACM has a very good RCS reduction performance. In order to better understand the performance of the ACM, Table 1 shows a comparison of the ACM with some previous designs, it is indicated that the thickness of the ACM is not very thick, but it has a significant advantage in the bandwidth expansion of RCS reduction, so it is very practical.

**Table 1:** Comparison of this work with previous designs.

References	RTH (*λ*_L_)	OFB (GHz)	FBW (%)	RBW
^29^	0.078	6.5–20.0	101.9	3.08
^30^	0.084	21–38	57.6	1.81
^31^	0.096	13.0–31.5	83.1	2.42
^32^	0.106	5.3–18.0	109.0	3.40
^33^	0.082	12.37–28.44	77.8	2.30
^34^	0.153	7.65–25.00	106.3	3.27
^35^	0.097	4.68–18.37	116.3	3.78
^3,6^	0.116	8.7–32.5	115.5	3.74
^3,7^	0.046	3.4–18.0	136.5	5.29
This work	0.096	7.4–45.5	144.1	6.15

*RTH* Relative thickness, *OFB* Operating frequency band, *FBW* Fractional bandwidth, *RBW* Ratio bandwith.

## Conclusions

In summary, a novel ACM was proposed based on an ultra-wideband lossy PCM in this paper, it can achieve ultra-wideband RCS reduction by means of both diffusion and absorption. The simulation and experimental results show that the ACM can achieve ultra-wideband diffusion-like scattering, and effectively reduce its RCS under normal incidence with arbitrary polarization in the ultra-wide frequency band from 7.4 to 45.5 GHz with a fractional bandwidth of 144.1%, moreover, an ultra-wideband RCS reduction can still be achieved under oblique incidence when the incident angle is increased to 45°. Compared with many previous works, the proposed ACM has a significant advantage in the bandwidth expansion of RCS reduction. In addition, it has the advantages of polarization-insensitivity and wide incident angle, so it is of great application values in radar stealth technology.

## Data Availability

The data generated during and/or analyzed during the current study are available from the corresponding author on reasonable request.
